# Intensification of Insulin Treatment With Insulin Degludec/Aspart in Type 2 Diabetic Patients: A 2-Year Real-World Experience

**DOI:** 10.3389/fcdhc.2022.783277

**Published:** 2022-07-26

**Authors:** Hatice Oner, Hatice Gizem Gunhan, Dilek Gogas Yavuz

**Affiliations:** ^1^ Department of Endocrinology and Metabolism, Marmara University School of Medicine, Istanbul, Turkey; ^2^ Department of Internal Medicine, Marmara University School of Medicine, Istanbul, Turkey

**Keywords:** Keywords: IDegAsp1, insulin2, degludec3, real world4, type 2 diabetes5, intensification6

## Abstract

**Aim:**

To evaluate the effects of insulin degludec/insulin aspart (IDegAsp) coformulation as an intensification of insulin treatment for glycemic control in patients with type 2 diabetes (T2D) in a long term real-world clinical setting.

**Materials and Methods:**

This retrospective non-interventional study, included 210 patients with T2D who to IDegAsp coformulation from prior insulin treatment in a tertiary endocrinology center between September 2017 and December 2019. The baseline data was taken as the index date and defined as the first IDegAsp prescription claim. Previous insulin treatment modalities, hemoglobin A1c (HbA1c), fasting plasma glucose (FPG), and body weight were recorded, respectively at the 3^rd^, 6^th^, 12^th^, and 24^th^ months of the IDegAsp treatment.

**Results:**

Out of the total 210 patients, 166 patients under insulin treatment switched to twice-daily IDegAsp treatment, 35 patients switched to once daily IDegAsp and twice premeal short-acting insulin regimen as a modified basal-bolus (BB) treatment, and nine patients commenced with once-daily IDegAsp treatment. HbA1c decreased from 9.2% ± 1.9% to 8.2% ± 1.6% in 6 months, 8.2% ± 1.7% in the first year, and 8.1% ± 1.6% in the second year of the therapy (*p*< 0.001). FPG decreased from 209.0 ± 85.0 mg/dL to 147.0 ± 62.6 mg/dL in the second year (*p*< 0.001). The required total daily dose of insulin increased in the second year of IDegAsp treatment compared to baseline. However, there was a borderline significance increase in IDegAsp requirement for the whole group at the two-year follow-up (*p* = 0.05). Patients who were administered twice daily IDegAsp injections required more total insulin in the first and second years due to added premeal short-acting insulin injections (*p* < 0.05). The frequency of patients with HbA1c < 7% was 31.8% in first year and 35.8% in second year under IDegAsp treatment.Insulin dose was de-escalated in 28.5% of the patients under BB treatment, while 15% under twice-daily IDegAsp required increased BB treatment.

**Conclusion:**

Intensification of insulin treatment with IDegAsp coformulation improved glycemic control in patients with T2D. The total daily insulin requirement increased but the IDegAsp requirement lightly increased at the two-year follow-up. Patients under BB treatment required de-escalation of insulin treatment.

## Introduction

Insulin degludec/insulin aspart (IDegAsp) is a fixed-ratio coformulation of insulin degludec (degludec, 70%) and insulin aspart (IAsp, 30%) in a single injection that provides both prandial and basal glycemic coverage (1–3); it is usually administered once- or twice-daily with the main meals. The basal component, Degludec,has a flat pharmacokinetic profile over 24 hours at a steady-state and provides a stable and long-lasting, glucose-lowering effect with less hypoglycemia than long-acting insulins (2–6).

IDegAsp coformulation is an insulin preparation whose dual action profile offers both prandial and basal glycemic coverage without the need for multiple injections (5, 7–9). Randomized clinical trials found that treatment intensification with IDegAsp BID reduces nocturnal severe hypoglycemic episodes while reaching the non-inferior limit mean reduction in HbA1c when compared to biphasic insulin Aspart 30 (8). IDegAsp coformulation provides similar glycemic efficacy and less nocturnal hypoglycemia in type 2 diabetic patients when compared to those using premix, basal-bolus (BB), and basal + oral antidiabetic drug combinations (10–12). A Japanese study showed that switching from insulin to IDegAsp coformulation significiantly improved glycemic control and reduced hypreducedng the at a 12 month follow-up of patients with type 2 diabetes (12). Premixed biphasic insulins can increase treatment compliance and achieve efficacy similar to that of basal-bolus therapy but with fewer injections (9, 11). Access to medication is remains a concern for people with diabetes (11).

Although robust data from randomized controlled trials have been published in the past, data from observational and real-world settings are scarce. A small retrospective study from India showed that IDegAsp coformulation reduced the insulin dose requirement when compared to premixed insulin (13).

IDegAsp coformulation has been available in Turkey since 2017 and reimbursed for insulin treatment intensifications in type 1 and type 2 diabetic patients. This study aimed to evaluate the effects of Insulin Degludec/Insulin Aspart (IDegAsp) as an intensification treatment for glycemic control in patients with type 2 diabetes (T2D) in a real-world clinical setting.

## Materials and Methods

### Patient Selection

This retrospective study included 210 patients T2D who underwent insulin treatment for at least one year and switched to IDegAsp coformulation as intensification treatment between September 2017 and December 2019 in the endocrinology outpatient clinic of Marmara University Pendik Research and Training Hospital.

Patients with the following conditions were excluded from the study: type 1 diabetes, active inflammatory and infectious disorders, active cancer treatment, end-stage renal and hepatic disease, female gestational diabetes, age under 18 years or over 85 years, insulin treatment for less than one year, acute hyperglycemia without insulin usage, and hospitalization.

The study protocol was approved by the local ethics committee of the Marmara University School of Medicine (09.2021.111). The study was performed according to the International Conference on Harmonization Guidelines for Good Clinical Practice and the Declaration of Helsinki.

### Clinical Evaluation

The following demographic, clinical, and laboratory data were recorded from the patients’ files: age; gender; duration of diabetes; duration of insulin treatment; daily insulin dose requirement; total, long, and short-acting insulin doses per day; daily IDegAsp dose requirement; injection frequencies, lipid parameters, oral antidiabetic medicine, and body weight.

All patients were undergoing analog insulin treatment, including Detemir, Glargine U100, Glargine U300 long-acting insulin, Lispro, Glulisine, and insulin Aspart as rapidly acting insulins.

Twice-daily IDegAsp regimen doses were calculated by converting between premix and basal insulin regiments, divided as two equal doses before morning and evening meals. IDegAsp OD/BID regimen was preferred for patients who reported nocturnal hypoglycemia and to reduce injection frequencies under basal-bolus treatment. IDegAsp dose calculations were performed for unit-to-unit basal and basal insulin requirements. Basal insulin regimens switched to IDegAsp OD if nocturnal hypoglycemia was a concern and if postprandial glucose control was needed.

Nocturnal hypoglycemia was defined as glycemia that occurred from midnight to 6 AM. Severe hypoglycemia was defined as an episode requiring another person to administer carbonhydrate, glucagon, or to take other corrective actions; plasma glucose concentrations may not be available.

The patients were organized into three categories according to the IDegAsp insulin regimen at the beginning of the study: once-daily IDegAsp (IDegAsp/OD) (*n* **=** 9, 4.3%), twice-daily IDegAsp (IDegAsp/DIB) (*n* **=** 166, 79%) and once-daily IDegAsp with two short-acting insulin injections (IDegAsp OD/BID) (*n* **=** 35, 16.7%).

In the two-year follow-up period, various changes in insulin treatment options categorized as de-escalation, intensification, and interchange to another type of insulin were recorded, at the two-year follow-up, and results were calculated by grouping patients in the relevant insulin regimen.

### Biochemical Parameters

Biochemical results in the 3^rd^, 6^th^, 12^th^, and 24^th^ months were recorded from the patients’ files. Fasting plasma glucose levels (FPG) were measured using an enzymatic UV test (hexokinase method); total cholesterol, HDL, and triglycerides were also analyzed using an enzymatic color method, whereas HbA1c was analyzed with high-performance liquid chromatography in Premier Hb9210 (Trinity Biotech, USA).

### Statistical Analysis

All the statistical analyses were conducted using SPSS (Statistical Package for the Social Sciences) version 20.0. Descriptive data was stated as frequencies (%) for categorical data, means, and standard deviations (SD) for continuous data with a normal distribution. Mann-Whitney U test, Kruskal-Wallis, and ANOVA tests were used to compare groups. A chi-square test was used to compare categorical data. The results were evaluated at a 95% confidence interval. The statistical significance level was accepted as *p*< 0.05, and the results were expressed as mean **±** SD values.

## Results

Out of all the 210 T2D patients, the mean age was 59.6 ± 10.5 years, and 131 (62.4%) patients were females. The mean duration of diabetes was 12.8 ± 7.1 years. The mean weight was 95.7 ± 19.6 kg, and HbA1c levels were 9.2% ± 1.9%. The mean daily insulin requirement was 62.0 ± 35.0 U.

During the inclusion initially 45.5% of the patients were receiving premixed insulin, 18.5% basal insulin, 3.3% basal plus, and 28% BB insulin.

At the time of initiation of after beginning IDegAsp treatment, 132 patients were treated with metformin (62.9%), 104 patients with DPP-4 inhibitors (49%), 13 patients with SGLT-2 inhibitors (6.2%), and 10 patients with sulfonylurea (4.8%). Eight patients were using GLP-1 analogue (3.8%), three patients were using alpha glucosidase inhibitors (1.4%), and one patient was using thiazolidinedione (0.5%). In the follow-up, metformin was initiated in 27 patients, SGLT-2 inhibitor in 15 patients, DPP-4 inhibitors in 31 patients, and thiazolidinedione in two patients. The drugs for patients using sulfonylurea and for patients using alpha glucosidase were discontinued. In addition, SGLT-2 inhibitor in five patients, the GLP-1 analogue in two patients, and the DPP-4 inhibitors in three patients were discontinued.

According to lack of follow-up or interchange to another insulin regimen, 132 patients in the first year and 81 patients in the second year were under IDegAsp treatment. **Table 1** shows insulin requirement and glycemic parameters during the follow-up period. HbA1c levels decreased after switching to IDegAsp treatment in the third month of the therapy (*p* < 0.001) and remained at the same level for two years period for all the groups (*p* < 0.001). Glycaemic parameters and insulin requirements for the IDegAsp regimen treated patients are shown in **Table 2**. In patients who switched to IDegAsp OD, IDegAsp BID, IDegAsp OD/BID regimen’s HbA1c levels decreased at the 3^rd^, 6^th^, 12^th^, and 24^th^ month of the therapy compared to before treatment levels.

**Table 1:** Type 2 DM patients’ insulin requirement and laboratory monitoring.

	Basal *n* :210	3. month *n* :193	6. month *n* :154	12. month *n* :132	24. month *n* :81	*p* value
**Weight (kg)**	95 ± 19.6	102 ± 20.3	103 ± 19.6	105 ± 22.5	106 ± 19.0	0.178
**FBG (mg/dL)**	209.0 ± 85.0	160.0 ± 64.0*	147.0 ± 63.2*	149.9 ± 61*	147.0 ± 62.6*	< 0.001
**HbA1c %**	9.2 ± 1.9	8.2 ± 1.5*	8.2 ± 1.6*	8.2 ± 1.7*	8.1 ± 1.6*	< 0.001
**Total-C (mg/dL)**	202.0 ± 53.5	194.0 ± 48.3	186.9 ± 49.5	180.9 ± 46.7*	168.8 ± 43.8*	< 0.001
**LDL-C (mg/dL)**	117 ± 41.9	114.7 ± 39.6	110.2 ± 42.9	103.5 ± 40.4*	94.8 ± 32.2*	0.0002
**HDL-C (mg/dL)**	44.7 ± 10.1	45 ± 11.0	44 ± 10.8	43 ± 10.0	44 ± 12.1	0.3663
**TG (mg/dL)**	223 ± 218.0	170 ± 96.8	165 ± 82.0	164 ± 111.0*	144 ± 86.3*	< 0.001
**Total insulin dose U/day**	62.0 ± 35.0	64.2 ± 35.5	67.1 ± 36.5	71.2 ± 37.8	76.6 ± 41.3*	0.009
**Total IDegAsp dose U/day**	54.8 ± 28.5	55.1 ± 28.2	58.4 ± 31.4	61.8 ± 32.3	65.3 ± 34.2	0.050

FPG, fasting plasma glucose; Total-C, total cholesterol; HDL-C, high-density cholesterol; LDL-C, low-density cholesterol; TG, triglyceride; *Compared to baseline.

**Table 2:** Insulin dose frequency and total and IDegAsp insulin requirement in Type 2 DM patients.

	Basal	3. month	6. month	12. month	24. month	*p-*value
**Once daily *n* (%)**	9 (4.3)	8 (4.1)	5 (3.2)	6 (4.6)	5 (6.2)	–
**Twice daily *n* (%)**	166 (79.1)	154 (79.8)	126 (81.8)	111 (83.9)	64 (79.0)	–
**Part of BB *n* (%)**	35 (16.7)	31 (16.1)	23 (14.9)	15 (11.5)	12 (14.8)	–
**Twice daily total insulin dose(U/day)**	62.2 ± 32.3	64.0 ± 33.4	69.8 ± 37.8	74.7 ± 37.6*	79.9 ± 42.6*	<0.05
**Twice daily IDegAsp dose (U/day)**	58.3 ± 27.3	57.8 ± 26.8	61.6 ± 28.0	66.0 ± 31.0	71.2 ± 32.8**	<0.05
**Part of BB total insulin dose (U/day)**	78.5 ± 45.0	75.2 ± 42.0	64.3 ± 25.9	66.4 ± 29.8	75.5 ± 28.8	0.781
**Part of BB IDegAsp dose (U/day)**	46.7 ± 30.5	44.1 ± 20.9	38.7 ± 15.5	37.1 ± 19.0	44.6 ± 19.1	0.520

*Compared to basal total insulin dose ** Compared to 3. monthIDegAsp dose.

During follow-up, there was a significant increase in the total daily insulin requirement significantly increased during follow-up only in the second year compared to baseline (*p* **=** 0.009). There was no statistically significant increase in the total daily insulin requirement of the patients who received IDegAsp as part of the BB treatment (*p* **=** 0.520). However, the IDegAsp requirement slightly increased during the two-year follow-up period in the whole group (*p* = 0.05). Patients who received twice-daily IDegAsp required 62.2 ± 32.3 U total daily insulin in the beginning, followed by 74.7 ± 37.6 U in the first year and 79.9 ± 42.6 U at the and of the second year (*p* < 0.05). The dosage in patients who received twice-daily IDegAsp was 58.3 ± 27.3 U at the baseline and 71.2 ± 32.8 at the end of the second year (*p* < 0.05) (**Table 2**). Similar glycemic control was not observed in the patients who received two doses as a part of basal-bolus therapy. Although HbA1c significantly decreased in patients using two doses of insulin (*p* < 0.01), the same decrease did not reach statistical significance in patients who participated in BB therapy (*p* = 0.236).

**Table 3** shows the insulin regimen and frequency in patients T2D under IDegAsp treatment at follow-up. Eighty one patients completed the two-year follow-up. Thirty-three patients switched to other insulin treatment regimens. Twenty-five patients in the first year and 39 patients in the second year lost of follow-up. Two of the patients (22.2%) using IDegAsp/OD were converted to twice-daily IDegAsp, and six patients (3.6%) using twice-daily IDegAsp were converted to IdegAsp OD/ BID treatment. Twenty-five of the patients who received twice daily IDegAsp switched to other insulin regimens, and ten of the patients who received IDegAsp as of BB insulin therapy were de-escalated.

**Table 3:** Insulin Regimen and Frequency in Type 2 Diabetes Patients Under IDegAsp Treatment.

	Once-daily	Twice daily	Part of basal-bolus	Total
**Initiation (n)**	9	166	35	210
**Intensification (n)**	2	6	–	8
**De-escalation (n)**	–	3	10	13
**Interchange (n)**	3	25	5	33

Patients with HbA1c less than 7% were, 15% at the third month, 24.6% at the sixth month,31.8% in the first year, and 35.8% in the second year. The weight of the patients did not significantly differ during the 2-year follow-up period (*p* = 0.178).

## Discussion

This retrospective, non-interventional study, monitored patients with T2D who switched from other insulin regimens to IDegAsp as part of routine clinical practice. Shifting to IDegAsp treatment for two years maintained glycemic control, increased total daily insulin but stable IDegAsp insulin dose requirements in comparison with the baseline data. IDegAsp treatment also reduced FPG an HbA1c values, which were consistent with the individual trials (14, 15).

Differences in glycemic control were statistically significant after switching from other insulin regimens to IDegAsp. A Japanese study also found that FPG also improved with IDegAsp versus BIAsp 30 in two treat-to-target RCTs of insulin-experienced patients with T2D (16, 17).

The mean baseline HbA1c was 9.2%, indicating that many patients in this study did not achieve optimal glycemic control with previous insulin regimens. This likely motivated their switch to the IDegAsp regimen. The decrease in HbA1c at 24 months (1.1%) observed here is similar to the targeted HbA1c reduction after 6 months of IDegAsp treatment in Japanese patients with T2D whose insulin levels were insufficiently controlled by previous therapies (1.4%) (16). RCT baseline HbA1c (8.3%) was lower in a previous study than in our study (9.2%), and the proportion of patients achieving HbA1c <7.0% was 52.5% (16), whereas our study found the same to be 33.3%. After it was reduced (8.2%) in the first three months, the median HbA1c value did not significantly decrease at the follow-up, indicating that IDegAsp treatment lowers blood glucose within a few months.

Our findings on fasting blood glucose levels are consistent with the current literature. Twenty-two patients, with T2D were administered premixed insulin for the first 2 months, followed by IDegAsp for 2 months (18); mean blood glucose levels (175.5 vs. 163.0 mg/dL; *p =* 0.004) were significantly lower in the IDegAsp phase when compared to the premixed phase measured before and after breakfast as well as before and after the evening meal (18). Here, the insulin dose requirement increased by 14 U during two-year follow-up period when compared to the baseline level (*p<*0.0095).

We also observed that there was no significant increase in, yet the total IDegAsp requirement did not significantly increase. The insulin requirement increased because the initial dose was reduced due to substituting previous insulin therapy with IDegAsp in all patients.

The IDegAsp coformulation simplifies patients’ lives and potentially improves glycemic control in the basal-bolus regimen when compared to concentrated insulin therapy (19). Administrating fewer injections may better overcome barriers to insulin intensification and reduce clinical inertia when compared with BB insulin regimens. The basal and bolus components in IDegAsp offer flexibility in administration timings and a greater opportunity for individualized therapy when compared to traditional premixed preparations (20).

This study enrolled more patients, and included a longer follow-up time than previous real-world studies in Turkey (21, 22). However, some key limitations should be considered when interpreting our study’s results. Firstly, whether the oral antidiabetic drug or IDegAsp coformulation affects glycemic control in patients who started with both oral antidiabetic drugs and the IDegAsp regimen remains unknown. Another limitation is that all the patients did not come to the follow-up until the second year, and some of them left the follow-up early in the sixth-month or the first year.

In conclusion, the usage of IDegAsp coformulation as a component of once-daily, twice-daily, and basal-bolus regimens over other insulin treatment options provides a therapeutic alternative to improve glycemic control, resulting in lowering of fasting blood glucose and HbA1c. Our findings provide important insights into the use of IDegAsp in a real-world setting in Turkey.

## Data Availability Statement

The original contributions presented in the study are included in the article/supplementary material. Further inquiries can be directed to the corresponding author.

## Ethics Statement

The studies involving human participants were reviewed and approved by Local ethics committee of the Marmara University School of Medicine. Written informed consent for participation was not required for this study in accordance with the national legislation and the institutional requirements.

## Author Contributions

HO, HG, and DY contributed equally to conception, design and writing of the manuscript. All authors revised the manuscript and approved the submitted version.

## Conflict of Interest

The authors declare that the research was conducted in the absence of any commercial or financial relationships that could be construed as a potential conflict of interest.

## Publisher’s Note

All claims expressed in this article are solely those of the authors and do not necessarily represent those of their affiliated organizations, or those of the publisher, the editors and the reviewers. Any product that may be evaluated in this article, or claim that may be made by its manufacturer, is not guaranteed or endorsed by the publisher.
